# One Drop | Mobile on iPhone and Apple Watch: An Evaluation of HbA1c Improvement Associated With Tracking Self-Care

**DOI:** 10.2196/mhealth.8781

**Published:** 2017-11-29

**Authors:** Chandra Y Osborn, Joost R van Ginkel, David G Marrero, David Rodbard, Brian Huddleston, Jeff Dachis

**Affiliations:** ^1^ Informed Data Systems Inc New York, NY United States; ^2^ Leiden University Leiden Netherlands; ^3^ The University of Arizona Health Sciences Tucson, AZ United States; ^4^ Biomedical Informatics Consultants LLC Potomac, MD United States; ^5^ Informed Data Systems Inc Austin, TX United States

**Keywords:** type 1 diabetes, type 2 diabetes, mobile health, mobile phone, smartwatch, glycated hemoglobin A1c, HbA1c, glycemic control, self-care behavior

## Abstract

**Background:**

The One Drop | Mobile app supports manual and passive (via HealthKit and One Drop’s glucose meter) tracking of self-care and glycated hemoglobin A_1c_ (HbA_1c_).

**Objective:**

We assessed the HbA_1c_ change of a sample of people with type 1 diabetes (T1D) or type 2 diabetes (T2D) using the One Drop | Mobile app on iPhone and Apple Watch, and tested relationships between self-care tracking with the app and HbA_1c_ change.

**Methods:**

In June 2017, we identified people with diabetes using the One Drop | Mobile app on iPhone and Apple Watch who entered two HbA_1c_ measurements in the app 60 to 365 days apart. We assessed the relationship between using the app and HbA_1c_ change.

**Results:**

Users had T1D (n=65) or T2D (n=191), were 22.7% (58/219) female, with diabetes for a mean 8.34 (SD 8.79) years, and tracked a mean 2176.35 (SD 3430.23) self-care activities between HbA_1c_ entries. There was a significant 1.36% or 14.9 mmol/mol HbA_1c_ reduction (F=62.60, *P*<.001) from the first (8.72%, 71.8 mmol/mol) to second HbA_1c_ (7.36%, 56.9 mmol/mol) measurement. Tracking carbohydrates was independently associated with greater HbA_1c_ improvement (all *P*<.01).

**Conclusions:**

Using One Drop | Mobile on iPhone and Apple Watch may favorably impact glycemic control.

## Introduction

The digital diabetes ecosystem is booming [1,2], with more than 1500 mobile apps supporting diabetes management [3], yet very few diabetes apps have been studied. For the few that have, they significantly reduce glycated hemoglobin A_1c_ (HbA_1c_) by an average 0.49% [4].

The HbA_1c_ measurement is the amount of hemoglobin in the blood with glucose attached to it. People are diagnosed with diabetes when their HbA_1c_ level is 6.5% or greater. An HbA_1c_ of 7.0% or greater puts people with diabetes at risk of developing macrovascular and microvascular complications, whereas a HbA_1c_ less than 7.0% or reducing HbA_1c_ by 1.0% prevents complications [5,6]. Diabetes self-care (eg, eating fewer carbohydrate grams, being more active, taking medications) improves HbA_1c_ levels.

Diabetes apps offer tracking of self-care and can educate and motivate people to better care for their health [1]. Together, the widely used diabetes apps rate highly in terms of functionality, aesthetics, and engagement [7]. Devices, sensors, wearables, and watches that passively collect data may bolster engagement. Passive data collection makes a more useful and less burdensome diabetes app [1,8]. Very few apps, however, offer manual and passive data collection from a mobile phone and a smartwatch, and no study to our knowledge has explored the health benefit of this type of digital solution.

The One Drop | Mobile app offers manual data entry, but also passive data collection via Apple’s HealthKit, Apple Watch, and the Bluetooth-enabled One Drop | Chrome glucose meter. We hypothesized that there would be a pre-post HbA_1c_ change among people with diabetes using the One Drop | Mobile app on an iPhone and Apple Watch. We also hypothesized self-care tracking with the app would be associated with HbA_1c_ change.

## Methods

### One Drop | Mobile: A Mobile Phone and Smartwatch App

The One Drop | Mobile app is free and available on iOS, WatchOS, and Android operating systems. One Drop users manually and passively (via HealthKit for iPhone and Apple Watch, Google Fit for Android mobile phones, and the Bluetooth-enabled One Drop | Chrome blood glucose meter) store and track blood glucose readings, medication doses, physical activity, and carbohydrates consumed. A built-in food library expedites carbohydrate tracking. A medication scheduler reminds users when a dose is due, and tracks doses upon confirmation. Statistics of tracked data are viewable on iPhone, Android, and Apple Watch.

Watch app users can enter data directly from their Watch, and view statistics of their data and monitor goal progress on the Watch face. They can get push notifications on their Watch, including medication reminders and motivational messages prompting and reinforcing self-care.

On the mobile phone app, users can view in-depth statistics of their data and track HbA_1c_ test results and body weight. An in-app “Newsfeed” delivers health tips, articles, infographics, and more. A “Community” section facilitates learning from, supporting, and receiving support from other users. The iPhone app has a “Notifications” inbox with data-driven insights, achievements, reminders, and support accumulated from other users.

### Procedures

On June 6, 2017, we identified people with type 1 (T1D) or type 2 diabetes (T2D) using the One Drop | Mobile app on an iPhone and Apple Watch who had manually entered at least two HbA_1c_ values in the app with HbA_1c_ test dates 60 to 365 days apart. We did not recruit participants. Instead, we analyzed data collected from real users who elected to use the One Drop | Mobile app on their mobile phone and smartwatch devices.

Users enter and store self-care and health data in the One Drop | Mobile app. All data exist in a secure server in the cloud. We characterized users with app-entered demographics (eg, gender, diabetes type). We tested their HbA_1c_ change (ie, self-reported HbA_1c_ collected in the app). We also tested if tracking self-care with the app (ie, the number of times food, activity, blood glucose, and medications were stored in the app between HbA_1c_ measurements) was associated with HbA_1c_ change.

All users agree to an end-user license agreement (EULA). In this agreement, it states that, as a user, you “grant One Drop a perpetual, transferrable, sublicensable, worldwide, nonexclusive, royalty-free license to reproduce, distribute, use, modify, remove, publish, transmit, publicly perform, publicly display, or create derivative works of Your User Content for any purpose without compensation to you, including for the purpose of promoting One Drop and the App, including after your account is cancelled or otherwise terminated.” It also states that, “One Drop...may track and report your activity inside of the App, including for analytics purposes.” The full EULA is available in the app and online.

### Measures

#### User Characteristics

Gender, diabetes type, and year of diagnosis are self-reported in the app. The difference between year of diagnosis and year of One Drop account creation determined years of diagnosed diabetes. Passively collected time zone data determined user location. User location was dichotomized as United States versus non-United States in analyses because few users outside the United States had entered two HbA_1c_ measurements required for inclusion.

#### Insulin Status

We reviewed medication names tracked and scheduled in the app to determine if a user was taking insulin or not.

#### Self-Care

We summed self-care data tracked between two HbA_1c_ entries (60-365 days apart), generating counts of blood glucose, food (carbohydrates), medications, activity, and the overall number of self-care entries tracked in the app during that time.

#### Glycemic Control

Test results and test dates of HbA_1c_ were self-reported in the app. Self-reported recall of a HbA_1c_ test is highly sensitive (99%) to medical records and claims data documenting an actual HbA_1c_ test [9]. A self-reported HbA_1c_ result is sensitive (79%) to a lab HbA_1c_ test result [10]. Further, we used mean blood glucose measured before the second HbA_1c_ test date to exclude invalid HbA_1c_ measurements and, subsequently, validate self-reported HbA_1c_ at that time point (see Analyses section).

We used HbA_1c_ test dates to calculate the number of days between HbA_1c_ entries. We divided 365 days by 12 months to get 30.42 (days per) month. We divided the number of days between HbA_1c_ entries by 30.42 (days per) month to get the number of months between HbA_1c_ measurements.

### Study Oversight

One Drop, Informed Data Systems Inc (IDS) received an exemption for institutional review board approval and a waiver of informed consent from Solutions IRB, an independent ethics review company (Little Rock, AR and Yarnell, AZ) to study all de-identified data owned by One Drop IDS. All One Drop | Mobile app users must actively agree to a EULA detailing data ownership and use.

### Analyses

All analyses were performed using SPSS version 23 (IBM Corp). Summary statistics characterized the sample. Mann-Whitney *U* tests were used for diabetes type differences with continuous variables, and chi-square tests for differences with dichotomous variables. One user with T1D selected “other” for gender. Because “other” gender was infrequently selected, we removed the “other” gender subgroup prior to testing diabetes type differences on gender.

To exclude invalid self-reported HbA_1c_ data, we used the formula HbA_1c_=(90-day mean blood glucose + 77.3)/35.6 [11] to compare self-reported HbA_1c_ to 90-day mean blood glucose, and excluded users with a greater or less than 2.0% difference (n=44 were excluded). Spearman rho correlations verified the relationship between self-reported HbA_1c_ and mean blood glucose consistent with prior research [12].

Two variables had missing data: gender (37/256, 14.4%) and duration of diagnosed diabetes (47/256, 18.3%). Multiple imputation corrected for missing data on these variables [13]. We used predictive mean matching [14,15] to impute 100 datasets.

Three mixed-effects repeated measures models tested mean HbA_1c_ differences. The first unadjusted model tested the effects of time, diabetes type, and the interaction of time by diabetes type. The second model tested these effects adjusted for a priori covariates: gender, location, years of diagnosed diabetes, and months between HbA_1c_ measurements. We restricted the third model to users with T2D and tested the time effect only adjusted for a priori covariates and insulin status.

Finally, four multiple regression models tested relationships between self-care tracking with the app and HbA_1c_ change. The first unadjusted model assessed the relationships between the amount of tracking by self-care type and HbA_1c_ change. The second model introduced diabetes type. The third model added a priori covariates. The fourth model included users with T2D only, a priori covariates, and insulin status.

## Results

Users (N=256) had T1D (n=65) or T2D (n=191), and were 22.7% (58/219) female, diagnosed with diabetes for a mean 8.34 (SD 8.79) years, and tracked a mean 2176.35 (SD 3430.23) self-care activities in the app between HbA_1c_ entries. Across each of four self-care types, the Shapiro-Wilk test statistic ranged from 0.22 to 0.86 (all *P*<.001), signifying a non-normal distribution. We dichotomized each self-care variable to tracked versus not tracked to satisfy assumptions of statistical tests.

Table 1 presents median and interquartile ranges, n (%), or mean and standard deviation with *P* values for diabetes type differences on observed variables before multiple imputation. Compared to users with T2D, users with T1D had diabetes for more years and entered more self-care data in the app between HbA_1c_ measurements, particularly blood glucose readings. Self-reported HbA_1c_ and 90-day mean blood glucose were strongly correlated (ρ=.75, *P*<.001), even when stratified by diabetes type (T1D: ρ=.84, *P*<.001; T2D: ρ=.72, *P*<.001). This is consistent with previous cohort studies reporting correlations varying from .71 to .86 [12].

In unadjusted and adjusted models, there was a significant 1.36% (14.9 mmol/mol) HbA_1c_ reduction (unadjusted and adjusted *F*=62.60, *P*<.001) during a median 4.06 (IQR 2.82) months (unadjusted: 8.26% [66.8 mmol/mol] to 6.90% [51.9 mmol/mol]; adjusted 8.72% [71.8 mmol/mol] to 7.36% [56.9 mmol/mol]). In the adjusted model, users with T1D had an average 0.41% (*F*=4.38, *P=*.04) higher HbA_1c_ than users with T2D, but there was no time by diabetes type interaction. After adjusting for a priori covariates and insulin status, users with T2D had a 1.27% (13.9 mmol/mol) HbA_1c_ reduction (*F*=364.50, *P*<.001; 8.16% [65.7 mmol/mol] to 6.89% [51.8 mmol/mol]).

Finally, using the app to track carbohydrates was associated with greater HbA_1c_ improvement even after adjusting for covariates and insulin status for users with T2D (all *P*<.01).

**Table 1:** Sample characteristics with tests of difference by diabetes type.

User characteristics		Total (N=256)	Type 1 diabetes (n=65)	Type 2 diabetes (n=191)	*P*^a^
**Gender, n (%)**					
	Male	161 (62.9)	40 (61.5)	121 (63.4)	.91
	Female	58 (22.7)	14 (21.5)	44 (23.0)	
**Location, n (%)**					
	United States	217 (84.8)	54 (83.1)	163 (85.4)	.66
	Europe	27 (10.5)	9 (13.8)	18 (9.4)	
	Asia	8 (3.1)	2 (3.1)	6 (3.1)	
	Pacific	2 (0.8)	0	2 (1.0)	
	Africa	2 (0.8)	0	2 (1.0)	
**Diabetes duration (years), mean (SD)**		8.3 (8.8)	13.3 (11.6)	7.1 (7.7)	<.001
Insulin status (yes), n (%)		136 (53.1)	65 (100)	71 (37.2)	<.001
**Self-care, n (%)**					
	App self-care entries	1439.5 (1809)	2055.0 (4264)	1318.0 (1463)	.002
	Food entries	17.0 (166)	15.0 (150)	18.0 (178)	.67
	Activity entries	628.5 (1049)	470.0 (1170)	664.0 (966)	.31
	Blood glucose entries	115.0 (243)	193.0 (567)	94.0 (210)	.02
	Medication entries	221.0 (452)	279.0 (3657)	207.0 (367)	.06
**Glycemic control**					
	Months between HbA_1c_ entries, median (IQR)	4.06 (2.82)	5.16 (4.29)	3.88 (2.66)	.003
	First HbA_1c_ (%), mean (SD)	8.23 (2.27)	8.31 (2.47)	8.20 (2.20)	.87
	Second HbA_1c_ (%), mean (SD)	6.80 (0.99)	7.09 (1.15)	6.70 (1.39)	.01

^a^ From chi-square or Mann-Whitney *U* tests.

## Discussion

We assessed the HbA_1c_ change of 256 people with diabetes using the One Drop | Mobile app on an iPhone and Apple Watch for up to one year. HbA_1c_ decreased by 1.36% (14.9 mmol/mol) in a median of approximately 4 months. Using the app to track carbohydrates was independently associated with HbA_1c_ improvement.

To our knowledge, this study is the first to evaluate the HbA_1c_ benefit of a tethered diabetes mobile phone and smartwatch app. One study asked people with T1D to use a phone and smartwatch app and give qualitative feedback [16]. Users appreciated entering and viewing data from their watch, the watch’s connectivity to their phone, and viewing reminders on their watch. One Drop | Mobile on Apple Watch delivers all three benefits and, based on our findings, may improve glycemic control.

There are study limitations. This is not a randomized controlled trial, preventing causal conclusions. The sample was self-selected, limiting generalizability. HbA_1c_ measurements were self-reported rather than assessed with a laboratory assay. Passively collected data are less prone to social desirability biases, but have their own reliability and validity issues [17]. The One Drop | Mobile app has features we did not evaluate or adjust for in our analyses. Finally, we do not know users’ age or socioeconomic status (eg, income, education, insurance status), preventing generalizability to all ages and socioeconomic groups.

Despite these limitations, people of all ages [18], race/ethnicities, and socioeconomic backgrounds [19] increasingly want to use smart devices to assist in the management of diabetes [20]. Research needs to critically evaluate diabetes apps, trackers, and smartwatches, especially as new devices enter the marketplace. Findings must be disseminated directly to consumers and to physicians who can assess these tools and make recommendations accordingly.

## Abbreviations

EULA: end-user license agreement
HbA_1c_: glycated hemoglobin A_1c_
IDS: Informed Data Systems
